# From Lipid Homeostasis to Differentiation: Old and New Functions of the Zinc Cluster Proteins Ecm22, Upc2, Sut1 and Sut2

**DOI:** 10.3390/ijms18040772

**Published:** 2017-04-05

**Authors:** Ifeoluwapo Matthew Joshua, Thomas Höfken

**Affiliations:** Division of Biosciences, Brunel University London, Uxbridge UB8 3PH, UK; ifeoluwapo.joshua@brunel.ac.uk

**Keywords:** zinc cluster protein, transcription factor, sterol uptake, sterol biosynthesis, anaerobic, filamentation, mating, budding yeast, *Candida*, antifungal

## Abstract

Zinc cluster proteins are a large family of transcriptional regulators with a wide range of biological functions. The zinc cluster proteins Ecm22, Upc2, Sut1 and Sut2 have initially been identified as regulators of sterol import in the budding yeast *Saccharomyces cerevisiae*. These proteins also control adaptations to anaerobic growth, sterol biosynthesis as well as filamentation and mating. Orthologs of these zinc cluster proteins have been identified in several species of *Candida*. Upc2 plays a critical role in antifungal resistance in these important human fungal pathogens. Upc2 is therefore an interesting potential target for novel antifungals. In this review we discuss the functions, mode of actions and regulation of Ecm22, Upc2, Sut1 and Sut2 in budding yeast and *Candida*.

## 1. Introduction

**Figure 1:**
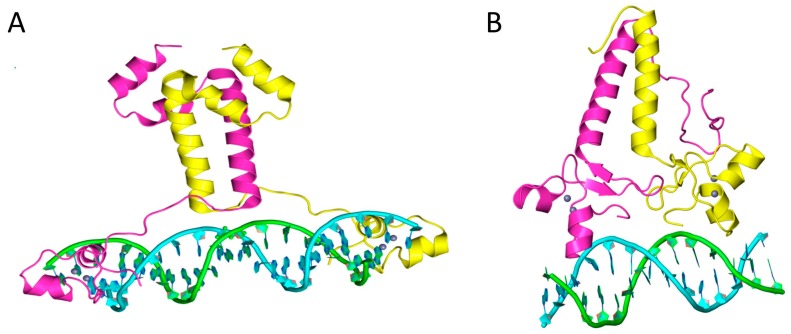
Structures of DNA-binding domains of zinc cluster proteins. (**A**) Crystal structure of the Gal4-DNA complex (PDB 1D66); and (**B**) crystal structure of the Ppr1 DNA-binding domain (PDB 1PYI). Both proteins form dimers. The two subunits are shown in yellow and pink. Grey spheres represent Zn^2+^ ions.

Zn(II)_2_Cys_6_ binuclear cluster proteins or just zinc cluster proteins are a large family of exclusively fungal transcriptional regulators [1,2]. The first studied member of this family was Gal4, which is one of the best characterized eukaryotic transcriptional activators. Zinc cluster proteins contain a well-conserved Cys-*X*_2_-Cys-*X*_6_-Cys-*X*_5-12_-Cys-*X*_2_-Cys-*X*_6–8_-Cys motif, which is part of a DNA-binding domain. The three-dimensional structure of Zn(II)_2_Cys_6_ cluster has been determined for several proteins including Gal4 and Ppr1 (Figure 1) [2,3,4]. In Gal4, Ppr1 and several other proteins, the first three cysteine residues of the motif bind to one Zn^2+^ ion and the next three cysteines conjugate a second Zn^2+^ ion. Binding of these zinc ions is necessary to form the compact structure of two short α helices separated by a loop. The Zn(II)_2_Cys_6_ cluster of most if not all members of the family seems to bind directly to DNA. In many proteins, the Zn(II)_2_Cys_6_ cluster is followed by a linker region that is also part of the DNA-binding domain [5] (Figure 1). This linker can be involved in DNA-binding specificity [6]. The Gal4 linker is extended and follows one DNA strand. In contrast, the Ppr1 linker forms an antiparallel β sheet and has no contact with DNA. In Gal4, Ppr1 and several other zinc cluster proteins the linker region is connected to a coiled-coil dimerization domain [5] (Figure 1). Some zinc cluster proteins might bind as monomers to DNA but the majority of proteins form dimers.

Zinc cluster proteins are best studied in the budding yeast *Saccharomyces cerevisiae* [2]. Its genome encodes 55 members of this family. Several zinc cluster proteins of the human fungal pathogen *Candida albicans* have also been well characterized. Sequencing of the genome allowed the identification of 77 putative zinc cluster proteins in this species [2]. Since this family of transcriptional regulators is quite large, it is not surprising that their members are involved in a wide range of functions from amino acid metabolism to multidrug resistance [2]. The zinc cluster proteins Sut1, Sut2, Upc2 and Ecm22 have initially been identified as important regulators of sterol uptake [7,8,9,10,11]. Later it was shown that these proteins also have other functions such as regulation of sterol biosynthesis and more recently differentiation. In this paper we review the functions of these zinc cluster proteins and describe the regulation of their activities. Since Sut1, Sut2, Upc2 and Ecm22 have first been characterized in budding yeast and because much of our understanding of their functions and regulation is largely derived from experiments using budding yeast, we first focus on this species in this review. We then highlight the role of *SUT1*, *SUT2*, *UPC2* and *ECM22* orthologs in the pathogenesis of several *Candida* species.

**Figure 2:**
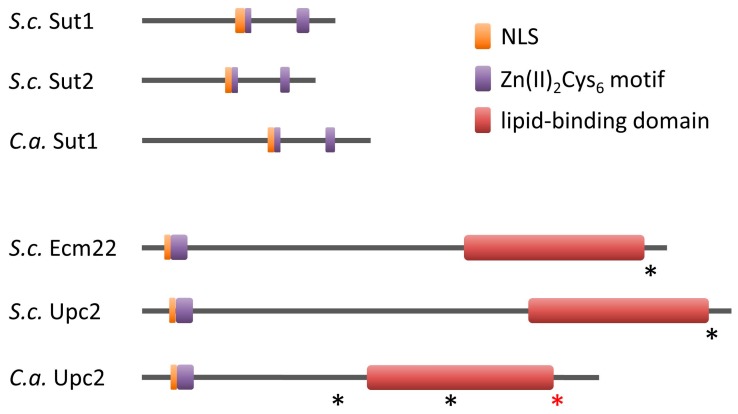
Domain structures of Ecm22, Upc2, Sut1 and Sut2. Shown are proteins from the budding yeast *S*. *cerevisiae* (*S.c*.) and *C*. *albicans* (*C.a*.). Asterisks denote gain-of-function mutations of Ecm22 and Upc2. The red asterisk of *C*. *albicans* Upc2 represents several distinct amino acid substitutions between residue 642 and 648.

Budding yeast *SUT1*/*SUT2* and *ECM22*/*UPC2* are paralogous pairs that probably arose from a whole-genome duplication of a budding yeast ancestor [10,11,12]. Ecm22 and Upc2 are not closely related to any other zinc cluster protein [13]. They have a perfect zinc cluster motif at the N-terminus as found in almost all zinc cluster proteins [5] (Figure 2). The DNA-binding domains of Ecm22 and Upc2 show some homology with the DNA-binding domain of Ppr1 and Gal4, respectively. Their three-dimensional structure might therefore also be similar (Figure 1).

Sut1/Sut2 are much smaller than Ecm22/Upc2 and the homology is rather low. Sut1 and Sut2 are unique among zinc cluster proteins as they do not have a 5–12 residue spacer in the centre of the Zn(II)_2_Cys_6_ cluster. Instead, the third and fourth cysteine are separated by a much larger spacer of over 60 residues [5,10]. It would therefore be interesting to determine their three-dimensional structure as this might be relevant to their function.

Since Ecm22, Upc2, Sut1 and Sut2 are transcription regulators, it is not surprising that nuclear localization signals (NLSs) have been predicted for all proteins (Figure 2). However, functionality has only been demonstrated for the Upc2 NLS [14]. Sut1 and Sut2 exclusively localize to the nucleus, whereas Ecm22 and Upc2 can shuttle between the cytoplasm and the nucleus [10,14,15,16,17].

## 2. Functions of Ecm22, Upc2, Sut1 and Sut2 in Budding Yeast

### 2.1. Regulation of Sterol Uptake

The budding yeast *Saccharomyces cerevisiae* is one of the few eukaryotes that can grow rapidly under both aerobic and strictly anaerobic conditions [18]. Yeast cells adapt to a lack of oxygen by inducing the expression of “anaerobic genes”. These genes encode for proteins that remodel sterol homeostasis, cell wall maintenance, respiration and carbohydrate metabolism.

Sterols are essential membrane lipids that are required for the structure and function of plasma membranes [19,20,21]. In fungi, the predominant sterol is ergosterol, the equivalent of mammalian cholesterol. In the presence of oxygen, yeast cells synthesize ergosterol and do not take up significant amounts of extracellular sterols [19]. This phenomenon, termed aerobic sterol exclusion, seems counterintuitive. Sterol biosynthesis is a complex process involving almost 30 enzymes and consumes large amounts of energy [21]. One may therefore expect that cells that are able to import sterols from the extracellular medium have a clear advantage. Aerobic exclusion might be a way for cells to ensure that only the best-fitting sterols accumulate in its membranes. In fact, cells with defects in the sterol biosynthesis pathway display pleiotropic defects such as impaired endocytosis, cell polarization, cell fusion and cell wall assembly [22,23,24,25,26]. Under anaerobic conditions, cells cannot synthesize ergosterol since this process requires oxygen, and cells import sterols, which are then required for viability [20]. Sut1, Sut2, Upc2 and Ecm22 all play important roles in the control of sterol uptake in the absence of oxygen.

Overexpression of either *SUT1* or *SUT2* induces sterol import, even under aerobic conditions [8,10]. However, the simultaneous deletion of *SUT1* and *SUT2* does not affect sterol import and growth under anaerobic conditions [10], presumably due to the presence of other regulators of sterol uptake such as Ecm22 and Upc2. The constitutively active mutant Upc2-1, in which glycine at position 888 near the C-terminus is altered to aspartic acid (G888D), also imports sterols in the presence of oxygen [7,9] (Figure 2). The C-terminal region containing the point mutation is highly similar between Ecm22 and Upc2 [11] (Figure 2). Introduction of the corresponding point mutation in Ecm22 (G790D) also results in increased sterol uptake [11].

The hyperactive Upc2-1 upregulates the expression of *AUS1* and *PDR11*, which encode two closely related members of the ATP-binding cassette (ABC) family of transporters [27]. Aus1 and Pdr11 form a complex in the plasma membrane and are together essential for sterol import [27,28,29]. *DAN1* is another anaerobic gene whose expression is induced by Upc2 [27,30]. *DAN1* encodes a cell wall mannoprotein that mediates sterol import [27,31]. It would be interesting to find out how cell wall proteins such as Dan1 and plasma membrane transporters such as Aus1 and Pdr11 function together in the uptake of sterols.

Little is known about the exact role of Ecm22 in sterol uptake. However, the anaerobic induction of *DAN1* seems to depend on both Ecm22 and Upc2 [32]. Sut1 stimulates the expression of *DAN1* and *AUS1*, like Upc2, but not of *PDR11* [17,31,33]. The underlying mechanisms of Sut2-mediated sterol uptake are unknown. Concomitant overexpression of *SUT1* and *SUT2* results in the same sterol uptake levels as overexpression of either *SUT1* or *SUT2* alone, suggesting that Sut1 and Sut2 may control the expression of the same or very similar targets [10]. Rather few target genes have been identified for Sut2 and all of them are also regulated by Sut1 [34,35]. However, none of the Sut2 target genes seems to be involved in sterol uptake.

### 2.2. General Adaptations to Anaerobic Conditions

Switching from sterol synthesis to sterol uptake is not the only adaptation to anaerobic conditions. Yeast cells also alter the expression of genes involved in cell wall maintenance and respiration [18]. Upc2 and Sut1 play important roles in these processes but are not the only regulators. Interestingly, budding yeast does not sense oxygen concentrations directly. Instead, transcription factors sense the levels of molecules that require oxygen for their synthesis, such as heme and ergosterol [18]. In the presence of oxygen, heme is synthesized and binds to Hap1, a transcriptional regulator of the zinc cluster protein family. Activation of Hap1 by heme induces the expression of aerobic genes including *ROX1*, which encodes a repressor of anaerobic genes. Rox1 binds to the promoter region of these genes often together with the general co-repressor Tup1-Cyc8 (also known as Tup1-Ssn6). Mot3, another DNA-binding protein, enhances Rox1 repression for some anaerobic genes. In the absence of oxygen, heme is no longer synthesized, which results in the deactivation of Hap1 and therefore reduced expression of *ROX1*. The loss of Rox1 repression then leads to activation of anaerobic genes. As described below, Rox1 also controls the transcription of *UPC2* and *SUT1*.

Sut1 and Upc2 regulate the expression of anaerobic genes. Over 100 anaerobic genes have an Upc2-binding site in their promoter [36]. This includes all but one member of the *PAU*/*DAN*/*TIR* group. The *DAN*/*TIR* family includes *DAN1*, *DAN4*, *TIP1* and *TIR1*-*4*. The related *PAU* family consists of 24 members including *PAU23* and *PAU24*, which were previously named *DAN2* and *DAN3*, respectively [37]. Expression of *PAU*/*DAN*/*TIR* genes is not or only partially controlled by Rox1 or Hap1 [30,36,38,39,40,41]. Upc2 and Sut1 both positively regulate the expression of over 10 *PAU*/*DAN*/*TIR* genes [27,30,31,41]. Rather little is known about these anaerobic proteins. Several of them are cell wall mannoproteins and seem to be involved in cell wall maintenance. As mentioned above, Dan1 mediates sterol uptake [27,31]. Complementation experiments in *Candida glabrata* using budding yeast *TIR3* suggest that *TIR3* might also have a role in sterol import [42]. There is no evidence that other *PAU*/*DAN*/*TIR* genes are also involved in sterol uptake. Interestingly, Upc2 not only upregulates the expression of *PAU*/*DAN*/*TIR* genes under anaerobic conditions; at the same time, Upc2 downregulates *CWP2*, a major aerobic counterpart of the *PAU*/*DAN*/*TIR* group [43]. Thus, Upc2 reciprocally controls the transcription of aerobic and anaerobic cell wall genes in the absence of oxygen.

Only few anaerobic target genes have been identified for Ecm22. It appears that Ecm22 induces at least some *PAU*/*DAN*/*TIR* genes, including *DAN1*, *PAU23* and *PAU24* [32,43].

Sut1 is also involved in remodelling respiratory metabolism in the absence of oxygen. It downregulates the expression of several genes with mitochondrial functions, which lowers the respiratory rate [31].

### 2.3. Control of Sterol Biosynthesis

Upc2 and Ecm22 do not only mediate sterol uptake; they also control sterol biosynthesis. Regulation of sterol levels occurs through feedback mechanisms at the transcriptional and post-transcriptional levels [44]. Lower cellular sterol levels increase the expression of *ERG* genes, which encode the enzymes that catalyse ergosterol biosynthesis, whereas higher sterol levels reduce *ERG* transcription. In the lab, sterol depletion is often achieved by using sterol biosynthesis inhibitors such as statins and azoles. Statins block HMG-CoA reductase, which catalyses an early step in sterol biosynthesis. Azoles are important antifungals that inhibit the enzyme Erg11, which functions at a later stage of ergosterol biosynthesis. Treatment with these drugs triggers the upregulation of *ERG* genes. This induction requires both Ecm22 and Upc2 [45]. Positive transcriptional regulation by Upc2 and Ecm22 has been demonstrated for many *ERG* genes [27,45,46]. Cells that lack Ecm22 and Upc2 have a very different sterol profile [47]. Ergosterol levels are strongly reduced and several intermediates, particularly of the later stage of the biosynthetic pathway, are enriched compared to the wild type. As a consequence, an *ECM22 UPC2* double deletion strain exhibits a much higher sensitivity to sterol biosynthesis inhibitors [45].

Interestingly, Upc2 also regulates the expression of *ARE1*, *ATF2* and *HES1*, which play roles in various aspects of sterol homeostasis [27]. Are1 catalyses the formation of steryl ester, the storage form of ergosterol [19,21]. Atf2 catalyses sterol acetylation, which seems to be important for sterol detoxification [19,20]. Hes1 (also known as Osh5) has ill-defined roles in sterol homeostasis. It is probably involved in sterol synthesis and intracellular sterol transport [48,49]. Unfortunately, these potentially interesting links have not been characterized yet.

Sut1 probably does not have a major role in sterol synthesis. *SUT1* overexpression has only a slight effect on the levels of sterol intermediates, but not of ergosterol [10]. Furthermore, expression of *ERG* genes is not regulated by Sut1 [31,35]. An involvement of Sut2 in sterol biosynthesis has not been reported.

In summary, Sut1, Sut2, Ecm22 and Upc2 all control sterol uptake in the absence of oxygen. Sut1 and Upc2 also regulate other adaptations to anaerobic conditions. Ecm22 and Upc2 regulate sterol biosynthesis. Several targets genes are overlapping for Ecm22 and Upc2 in sterol biosynthesis, and for Sut1 and Upc2 for the adaptations to anaerobic conditions. Ecm22, Upc2, Sut1 and Sut2 are largely positive transcriptional regulators in these processes.

## 3. Regulatory Mechanisms

Interestingly, the regulatory mechanisms for the processes described above seem to be connected. Under optimal conditions some Ecm22 and Upc2 bind to *ERG* promoters, which is important for uninduced expression of *ERG* genes [45]. When sterol levels drop following treatment with sterol biosynthesis inhibitors, Ecm22 is released from *ERG* promoters, a process that involves Mot3 [32,50]. At the same time, *UPC2* expression is upregulated and binding of Upc2 to *ERG* promoters strongly increases, which results in *ERG* induction [50,51]. This increase in *UPC2* expression can be explained by autoregulation. The *UPC2* promoter contains Upc2-binding sites and positive control of its own transcription has been demonstrated for Upc2 [27,30,52].

**Figure 3:**
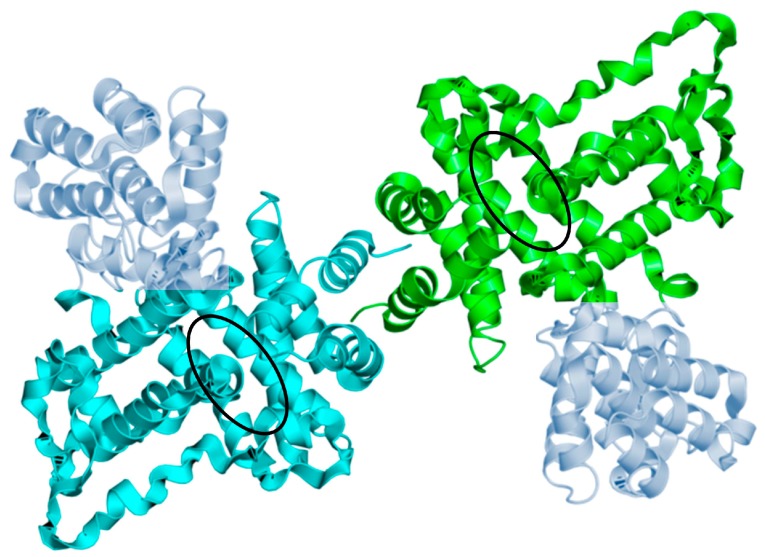
Crystal structure of the Upc2 lipid-binding domain. The lipid-binding domain forms dimers shown in green and turquoise (PDB 4N9N). To improve diffraction quality for crystallographic studies, a T4 lysozyme has been inserted between helix 5 and 6 (shown in light blue) [14]. The hydrophobic pockets are shown by the black ellipses.

**Figure 4:**
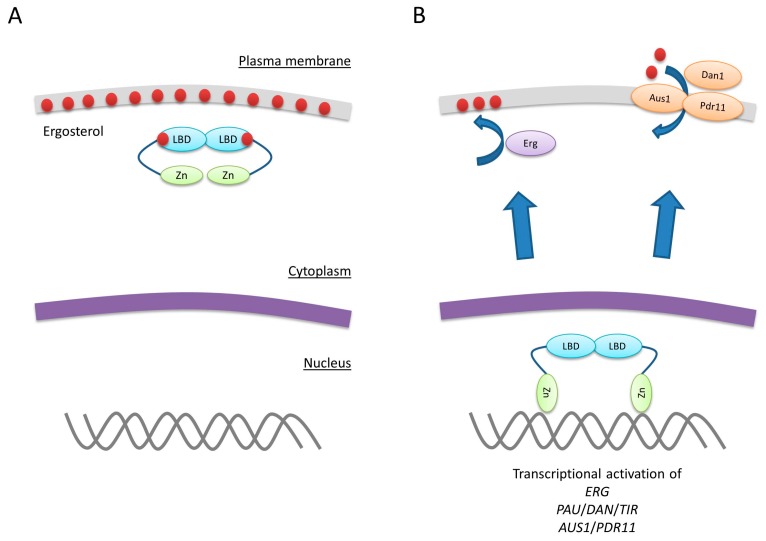
Regulation of Upc2 activity through lipid sensing. (**A**) When ergosterol (red spheres) levels are high it binds to the lipid-binding domain (LBD) of Upc2 and keeps the protein in the cytoplasm, possibly because the lipid-binding domain masks the NLS that lies adjacent to the Zn(II)_2_Cys_6_ motif (Figure 2) (Zn). (**B**) When sterol levels drop ergosterol no longer binds to Upc2. This might trigger a conformational change of the protein, which results in a translocation to the nucleus. In the nucleus Upc2 induces genes involved in sterol biosynthesis, sterol uptake and general adaptations to anaerobic conditions.

Control of *UPC2* transcription is an important regulatory mechanism for sterol biosynthesis. However, the recent determination of the structure of the C-terminal domain of Upc2 revealed an additional control mechanism [14]. The C-terminal region of Upc2 serves as a lipid-binding domain (Figure 2). When ergosterol is plentiful, this domain extracts sterol from the plasma membrane. This is a highly specific process. The lipid-binding domain can extract ergosterol and the related dehydroergosterol but not other sterols such as cholesterol. The lipid-binding domain is a novel fold not present in any other protein. Eleven α helices and connecting loops form a hydrophobic pocket for ergosterol in the core of the protein (Figure 3). The lipid-binding domain is also important for Upc2 dimerization as two α helices of each subunit form a hydrophobic dimer interface (Figure 3). Under sterol-rich conditions, Upc2 binds ergosterol and localizes predominantly to the cytoplasm, possibly because the lipid-binding domain masks the NLS [14] (Figure 4). When sterol levels drop, ergosterol dissociates from Upc2, which could result in a conformational change that exposes the NLS and allows the protein to translocate to the nucleus, where it induces expression of *ERG* genes [14,16]. The analysis of the lipid-binding domain also provides a mechanism for the gain-of-function mutant *UPC2-1* [9]. The G888D mutation lies near the lipid-binding domain and disrupts sterol binding [14] (Figure 2). The mutant protein can only be found in the nucleus, where it induces gene expression.

Ecm22 also possesses a lipid-binding domain that extracts sterols from membranes and translocates to the nucleus in response to sterol depletion [14,16]. Nevertheless, Ecm22 only seems to play a minor role in *ERG* gene induction.

**Figure 5:**
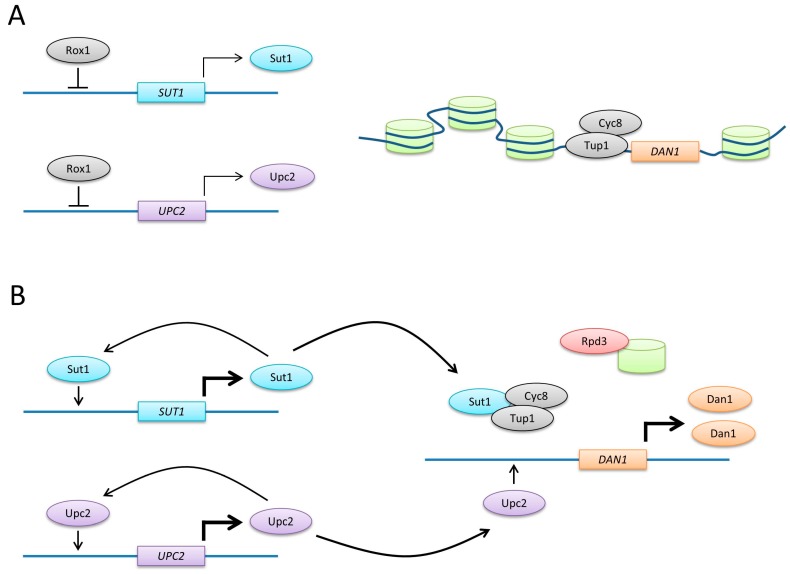
Transcriptional regulation of *DAN1*. (**A**) *SUT1* and *UPC2* are partially repressed by Rox1 in the presence of oxygen. The anaerobic gene *DAN1* is not expressed due to chromatin-mediated repression. (**B**) Under anaerobic conditions, nucleosomes are released from the *DAN1* promoter following histone deacetylation by Rpd3. Rox1 repression is lifted, which results in increased expression of *UPC2* and *SUT1*. Upc2 induces *DAN1* expression through direct binding with the promoter, whereas Sut1 might inactivate the co-repressor complex Cyc8–Tup1. Experiments that led to this model have largely been done with *DAN1*. However, these mechanisms might also apply to the regulation of other anaerobic genes.

The expression of *ERG* genes in response to sterol depletion and the adaptation to anaerobic growth are regulated by Upc2 and Ecm22 through similar mechanisms. Sterols cannot be synthesized in the absence of oxygen. This reduction of sterol levels serves as the primary signal for the induction of several *PAU*/*DAN*/*TIR* genes by Upc2 and Ecm22 [32]. It therefore seems likely that Upc2 also translocates from the cytoplasm to the nucleus when oxygen levels drop. In addition, anaerobic genes are induced through increasing Upc2 protein levels. In the presence of oxygen, *UPC2* is expressed but partially repressed by Rox1 [36] (Figure 5). Under anaerobic conditions, *UPC2* expression increases due to the loss of Rox1 repression and autoregulation [27,30,32,36].

The expression patterns of Sut1 and Upc2 are quite similar. Early Northern blotting experiments suggested that *SUT1* is not expressed under aerobic conditions [8]. However, later real-time PCR and immunoblotting experiments showed that mRNA and protein are aerobically expressed and induced under anaerobic conditions, as suggested earlier [8,34,53]. Rox1 partially represses *SUT1* in the presence of oxygen [8] (Figure 5). *SUT1* expression is also autoregulated. Several Sut1 binding sites have been identified in the *SUT1* promoter, and Sut1 positively regulates its own transcription [34,54].

*SUT2* induction in response to the lack of oxygen has been reported, but the underlying regulatory mechanisms are unknown [55].

The anaerobic induction of *PAU*/*DAN*/*TIR* by Upc2 requires the histone deacetylase Rpd3 [56]. An Rpd3 complex that includes Pho23, Sap30, Sds3, Sin3 and Ume1 targets the promoter of DAN1 and probably other *PAU*/*DAN*/*TIR* genes. Following promoter binding under anaerobic conditions, Rpd3 deacetylates the N-termini of histones H3 and H4. This results in the removal of nucleosomes from *DAN1*, which is necessary for Upc2 binding to the *DAN1* promoter (Figure 5).

Some of the *PAU*/*DAN*/*TIR* genes and *AUS1* are upregulated under anaerobic conditions by both Upc2 and Sut1 [27,30,31,33]. However, the mechanisms of transcriptional activation seem to be different. *PAU/DAN*/*TIR* genes, as well as *AUS1* and *PDR11*, possess Upc2-binding sites in their promoters and Upc2 acts through these sites [27,30,36,57]. In contrast, it has been suggested that Sut1 does not bind directly to the *DAN1* promoter [33]. Furthermore, a genome-wide screen that identified several Sut1 targets did not pick up any anaerobic genes [58], and no Sut1 binding site have been identified in the promoter regions of *DAN1* and *AUS1* [54,58]. Sut1 might act instead through the general co-repressor Tup1-Cyc8 [33]. Relieving this repression would result in increased transcription of *DAN1* (Figure 5).

Sut1 is also regulated by the p21-activated kinases (PAKs) Ste20, Cla4 and Skm1 [17]. PAKs are important signalling molecules that are activated by the Rho GTPase Cdc42 [59]. PAKs can translocate to the nucleus, where they bind to Sut1 and downregulate the expression of *AUS1* and *DAN1* in a Sut1-dependent manner. This results in reduced sterol uptake [17]. Notably, PAKs also regulate sterol synthesis and storage, suggesting that the regulation of sterol homeostasis is important for functions of PAKs such as cell polarization, filamentation and hyperosmotic shock [60]. Such links have indeed been reported [22,24,25,26,35,61].

## 4. Adaptations to Changing Environmental Conditions

Sterol homeostasis is not only important for normal cell growth under aerobic and anaerobic conditions. Regulation of sterol biosynthesis also plays a critical role in the adaptation to changing environmental conditions. Sterol levels decrease, for example, in response to hyperosmotic stress but increase during filamentation [35,61].

Hyperosmotic stress, which can be caused by high extracellular salt concentrations, triggers the high osmolarity glycerol (HOG) pathway [62]. Plasma membrane-bound receptors sense an increase in extracellular osmolarity, which leads to the activation of the mitogen-activated protein (MAP) kinase Hog1. Through phosphorylation of various substrates, Hog1 induces adaptations to hyperosmotic stress such as the synthesis of the osmolyte glycerol [62]. Activated Hog1 also increases the expression of *MOT3* and, to a lesser extent, *ROX1* [61]. Mot3 and Rox1 mediate the transcriptional repression of several *ERG* genes. These two repressors also lower *ECM22* expression, which contributes to *ERG* repression as well. This downregulation of several *ERG* genes results in reduced ergosterol levels. However, since some but not all *ERG* genes are repressed, sterol levels may not be simply lowered. It could be that the membrane sterol composition is modulated. While the alterations of the sterol profile seem to be an important adaptation to hyperosmotic stress, it is unfortunately not known how exactly cells are protected.

Ecm22 and Upc2 also positively regulate filamentation through increased sterol synthesis [35]. Filamentation occurs when cells grow on a semisolid medium with limited nutrients [63]. Under these conditions, cells become more elongated and do not separate following division. Furthermore, cells attach to and penetrate the medium they are growing on. Filamentous growth is therefore considered to represent a foraging mechanism. Ecm22 and Upc2 are both required for filamentation [35]. Several *ERG* genes are induced in an Ecm22- and Upc2-dependent manner when cells switch from the yeast form to the filamentous form. *ERG* genes seem to play a crucial role in this developmental transition since they are essential for filamentation. In addition, *ERG* expression is not only controlled by Ecm22 and Upc2 but also by Ste12, Phd1, Mga1 and Flo8, which are key transcriptional regulators of filamentation [64]. As a consequence, the ergosterol concentration increases during filamentation. Similar to the altered sterol levels in response to hyperosmotic stress, it remains unknown why exactly changes of sterol concentrations are required during filamentation.

Ecm22 and Upc2 not only control *ERG* genes but also upregulate *FHN1*, *NPR1* and *PRR2* expression during filamentation. *FHN1*, *NPR1* and *PRR2* play important roles in filamentous growth.

Sut1 and Sut2 are also key regulators of filamentation but, in contrast to Ecm22 and Upc2, they inhibit filamentation [15,34,35]. *GAT2*, *HAP4*, *MGA1*, *MSN4*, *NCE102*, *NPR1*, *PRR2*, *RHO3* and *RHO5* have been identified as targets of both Sut1 and Sut2 [34,35,58,65]. All of them are either essential for filamentation or at least play an important role in this process [34,35,66,67]. The expression of these genes, with the exception of *MGA1*, is upregulated when cells switch from the yeast form to the filamentous form [34,35]. The Sut1/Sut2 target genes seem to play a crucial role in filamentation because like *ERG* genes they also have binding sites for the filamentation master regulators Flo8, Mga1, Phd1, Ste12, Sok2 and Tec1 [64]. Sut1 and Sut2 partially repress the expression of *GAT2*, *HAP4*, *MGA1*, *MSN4*, *NCE102*, *NPR1*, *PRR2*, *RHO3* and *RHO5* when nutrients are plentiful [34,35,65]. When cells grow on a semisolid substratum with limited nutrients, this repression is lifted and results in an induction of the Sut1/Sut2 targets. Combined activity of the corresponding proteins might then trigger filamentation.

The mechanisms of transcriptional regulation by Sut1 seem to be different for filamentation and adaptation to anaerobic growth. It has been suggested that induction of *DAN1* in the absence of oxygen is not achieved through direct interaction of Sut1 with DNA but rather through inactivation of the general co-repressor Tup1-Cyc8 [33]. In contrast, Sut1 binds directly to DNA to regulate the expression of targets involved in filamentation, and DNA-binding motifs have been identified for Sut1 [54,58].

**Figure 6:**
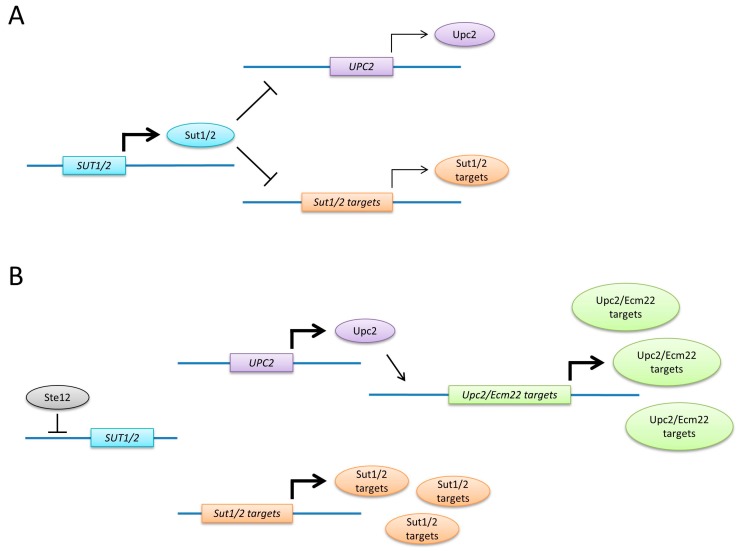
Transcriptional regulation of filamentation. (**A**) Under nutrient-rich conditions, *SUT1* and *SUT2* are expressed and partially repress *UPC2* and target genes that have a role in filamentation; (**B**) when cells grow on a semisolid medium with limited nutrients, Ste12 becomes activated, which represses *SUT1* and *SUT2*. This results in induction of the Sut1/Sut2 target genes including *UPC2*, which in turn leads to increased expression of Ecm22/Upc2 targets. The combined action of increased levels of Sut1/Sut2 and Ecm22/Upc2 targets might then trigger a switch to filamentous growth. Arrows indicate induction of gene expression, T-bars represent repression.

Sut1, Sut2, Ecm22 and Upc2 are regulated through a transcriptional network during filamentation (Figure 6). When cells sense nutrient deprivation and a semisolid substratum, the transcription factor Ste12 becomes activated [63]. Ste12 downregulates the expression of *SUT1* and *SUT2*, which results in induction of Sut1/Sut2 targets [34,35]. Among these targets is *UPC2*, whose expression increases during filamentation [35]. This in turn leads to the upregulation of Upc2 targets (*FHN1*, *NPR1*, *PRR2* and *ERG* genes).

Sut1, Sut2, Ecm22 and Upc2 also regulate mating. There are two distinct haploid cell types in budding yeast, termed a cells and α cells [68,69]. The two cell types secrete different pheromones to elicit a mating response in cells of the opposite cell type. Pheromone signalling triggers a G_1_ arrest and formation of a mating projection, which is required for subsequent cell fusion. Sut1 and Sut2 play a positive role in mating [65], which is achieved through repression of *NCE102*, *PRR2* and *RHO5*. The corresponding proteins have important roles in filamentation, as described above. They also inhibit mating and are therefore downregulated in response to pheromone treatment [65,70]. It therefore seems that Sut1 and Sut2 control the fate of a cell through the same set of genes. *NCE102*, *PRR2* and *RHO5* expression is upregulated under conditions that trigger filamentation due to the loss of Sut1/Sut2 repression. When cells are exposed to pheromones, transcription of *NCE102*, *PRR2* and *RHO5* is repressed, which allows cells to mate. We have recently found that Ecm22 and Upc2 also regulate mating by a mechanism that is independent of the regulation of *NCE102*, *PRR2*, *RHO5* and *ERG* gene expression (T. Höfken, manuscript in preparation).

Taken together, Ecm22, Upc2, Sut1 and Sut2 all regulate cell fate decisions such as filamentation and mating. In contrast to sterol uptake and other adaptations to anaerobic growth, Ecm22, Upc2, Sut1 and Sut2 control different sets of genes (with the exception of *PRR2* and *NPR1*, which are regulated by all four zinc cluster proteins) and they also have opposing roles. Sut1 and Sut2 are negative regulators of filamentation, whereas Ecm22/Upc2 induces filamentous growth.

## 5. Orthologs of *ECM22*, *UPC2*, *SUT1* and *SUT2* in Other Species

Orthologs of *ECM22*, *UPC2*, *SUT1* and *SUT2* exist in other fungi. However, *UPC2*/*ECM22* seem to be restricted to species of the Saccharomycotina, a lineage within the Ascomycetes [13]. The Saccharomycotina include the budding yeast *S*. *cerevisiae* and all *Candida* species such as *C*. *albicans*, *C*. *glabrata*, *C*. *parapsilosis* and *C*. *tropicalis*. In other fungi and higher eukaryotes, some functions of Ecm22 and Upc2, including regulation of sterol synthesis and uptake, and adaptation to low oxygen levels, are carried out by sterol regulatory element-binding proteins (SREBPs) [44,71]. Ecm22/Upc2 and Sut1/Sut2 are paralogous pairs that arose from a whole genome duplication of a common ancestor of budding yeast and *C*. *glabrata* [10,11,12,13]. The other species listed here are more distant relatives of budding yeast that did not undergo this duplication event. It is therefore not surprising that *UPC2*/*ECM22* have two orthologs in *C*. *glabrata* but only one in *C*. *albicans* [72,73,74].

The existence of orthologs raises the question of whether the functions and molecular mechanisms of the corresponding proteins are the same in budding yeast and other species. Rewiring of gene regulatory networks is an important mechanism of evolutionary adaptation [75]. It has, for example, recently been shown that the zinc cluster protein Ppr1 regulates pyrimidine biosynthesis in budding yeast but allantoin catabolism in *C*. *albicans* [76]. Considering the very different environment budding yeast and *Candida* live in, major rewiring can be expected. Nevertheless, it seems that at least some processes, such as the regulation of sterol biosynthesis by Upc2, are relatively well conserved among the species described here. Upc2-binding sites can, for example, be found in most *ERG* genes of species of the Saccharomycotina lineage but not outside of this group [13]. Furthermore, the Upc2 lipid-binding domain is highly conserved among species of the Saccharomycotina, suggesting that these species sense sterol levels through the same mechanism [14].

### 5.1. Upc2 and Sut1 in C. albicans

*C*. *albicans* is an important human fungal pathogen [77,78]. It is a commensal that lives in the gastrointestinal and genitourinary tracts of healthy individuals. In immunocompromised patients, *Candida* cells can enter the bloodstream and invade virtually every tissue, which results in a very high mortality rate. These systemic *C*. *albicans* infections are usually treated with azoles, which inhibit Erg11, an enzyme of ergosterol biosynthesis. Since azoles are fungistatic and not funcicidal, cells can develop resistance, which is a clinical problem. *UPC2*, the sole ortholog of budding yeast *UPC2* and *ECM22* in *C*. *albicans*, is involved in azole resistance [72,73].

*C*. *albicans* Upc2 plays an important role in sterol biosynthesis. It directly binds to *ERG* promoters and induces *ERG* transcription in response to sterol depletion [52,72,73,79]. *UPC2* expression is also autoregulated [73,80,81]. Gain-of-function mutants of *UPC2* have been identified in several azole-resistant clinical isolates [82,83,84,85]. Almost all of them encode amino acid substitutions in or near the lipid-binding domain of Upc2 (Figure 2). These mutations could prevent binding of ergosterol to the hydrophobic pocket and, as described for the G888D mutation of budding yeast Upc2, render the protein constitutively active in the nucleus [14]. The gain-of-function mutations cause increased expression of *ERG11* and other *ERG* genes, which results in higher ergosterols levels and therefore reduced azole susceptibility [82,83,84,85].

Upc2 also positively regulates the expression of *MDR1*, which encodes a multidrug transporter of the major facilitator family that exports azoles [79,82,85,86]. Even though Upc2 seems to play a minor role in this process, it could contribute to azole resistance.

The gain-of-function mutations in Upc2 provide an advantage in the presence of azoles. At the same time, these mutants display decreased fitness, and as a consequence are also less virulent [87,88]. However, the role of *UPC2* in virulence is not entirely clear. One study has reported that *UPC2* deletion results in increased kidney colonization, whereas others have observed the opposite effect for the *UPC2* deletion [88,89].

Upc2 plays a critical role in the adaptation to anaerobic conditions. Deletion of *UPC2* has been reported to either be lethal or result in severely impaired growth in the absence of oxygen [73,90]. Furthermore, *UPC2* expression is induced in response to anaerobic conditions [79,80]. Notably, hypoxia (1% oxygen) is one of the environmental signals that trigger filamentation in *C*. *albicans*. Hypoxia-induced filamentation requires *UPC2*, which is interesting because filamentation plays important roles in host cell adherence, tissue invasion and virulence [78,91,92]. It has also been reported that a *UPC2* gain-of-function mutation reduces filamentation [88]. The role of *UPC2* in this morphological switch is therefore not entirely clear.

The absence of *UPC2* orthologs in humans, the role of Upc2 in azole resistance and potentially other important roles in *Candida* pathogenesis make Upc2 an interesting target for novel antifungal drugs [93].

Recently, a role for *SUT1*, the sole ortholog of budding yeast *SUT1* and *SUT2*, has been established in *C*. *albicans* virulence [94]. A strain lacking *SUT1* exhibits reduced kidney colonization and is defective in virulence. Sut1 is required for zinc acquisition, which is essential for infection. Sut1 regulates this process by inducing the expression of the transcription factor gene *ZAP1*. Zap1 in turn activates the expression of zinc acquisition genes. Interestingly, Upc2 binds directly to the *SUT1* promoter and positively regulates *SUT1* expression [79]. It would be interesting to further characterize this link and find out whether this constitutes a regulatory network of zinc cluster proteins during infection.

### 5.2. UPC2 in Other Candida Species

Other *Candida* species can also cause systemic infections [77]. *C. glabrata* is now emerging as a more common pathogen because it is less susceptible to azoles than *C*. *albicans*. The fact that *C*. *glabrata* can import sterols under aerobic conditions in response to sterol-containing serum and bile could be an explanation for its reduced azole sensitivity [95,96,97]. Serum also induces expression of several *ERG* genes. *UPC2A* and *UPC2B*, the two orthologs of budding yeast *ECM22* and *UPC2* have roles in sterol homeostasis [74,98]. They are both required for serum-induced sterol import. Upc2A upregulates the expression of several genes in this process including *UPC2B* and *AUS1*, the sole ortholog of budding *AUS1* and *PDR11* [74,98,99]. Expression of *TIR3*, the ortholog of budding yeast *TIR3*, is also induced by Upc2A [42]. The plasma membrane protein Aus1 and the cell wall protein Tir3 cooperate in sterol uptake [42,99].

*UPC2* orthologs also have important functions in azole resistance in *C*. *parapsilosis* and *C*. *tropicalis* [100,101,102,103,104]. *UPC2* has been found to be upregulated and to contain mutations in azole-resistant clinical isolates. However, there are also important differences. The Upc2 mutations found in azole-resistant strains lie in the centre of the protein and not in or near the C-terminal of the lipid-binding domain of Upc2, as in *C*. *albicans* [103,104]. The effect of these mutations on the proteins is therefore not clear.

## 6. Conclusions

Initially, it seemed that cellular sterol homeostasis was the sole function of the zinc cluster proteins Ecm22, Sut1, Sut2 and Upc2. Homeostasis was achieved by regulating the transcription of sterol biosynthesis genes when sterol levels drop, and inducing the expression of sterol uptake genes in the absence of oxygen. More recently, it became evident that sterol levels need to be altered to cope with changing environmental conditions such as hyperosmotic stress. It was also found that Ecm22, Sut1, Sut2 and Upc2 have roles in other biological processes beyond sterol homeostasis such as mating and filamentation. More of these rather unexpected functions might be revealed in the future. Not surprisingly, much fundamental research has been done in budding yeast. However, since the importance of Upc2 and, to a lesser extent, Sut1, in *Candida* pathogenesis has become clearer, more research will probably focus on these proteins in *Candida*. Since zinc cluster proteins are fungal-specific, they are interesting targets for novel antifungals.

## References

[1] Todd R.B., Andrianopoulos A. (1997). Evolution of a fungal regulatory gene family: The Zn(II)2Cys6 binuclear cluster DNA binding motif. Fungal Genet. Biol..

[2] MacPherson S., Larochelle M., Turcotte B. (2006). A fungal family of transcriptional regulators: The zinc cluster proteins. Microbiol. Mol. Biol. Rev..

[3] Marmorstein R., Carey M., Ptashne M., Harrison S.C. (1992). DNA recognition by GAL4: Structure of a protein-DNA complex. Nature.

[4] Marmorstein R., Harrison S.C. (1994). Crystal structure of a PPR1-DNA complex: DNA recognition by proteins containing a Zn_2_Cys_6_ binuclear cluster. Genes Dev..

[5] Schjerling P., Holmberg S. (1996). Comparative amino acid sequence analysis of the C_6_ zinc cluster family of transcriptional regulators. Nucleic Acids Res..

[6] Reece R.J., Ptashne M. (1993). Determinants of binding-site specificity among yeast C_6_ zinc cluster proteins. Science.

[7] Lewis T.L., Keesler G.A., Fenner G.P., Parks L.W. (1988). Pleiotropic mutations in *Saccharomyces cerevisiae* affecting sterol uptake and metabolism. Yeast.

[8] Bourot S., Karst F. (1995). Isolation and characterization of the *Saccharomyces cerevisiae SUT1* gene involved in sterol uptake. Gene.

[9] Crowley J.H., Leak F.W., Shianna K.V., Tove S., Parks L.W. (1998). A mutation in a purported regulatory gene affects control of sterol uptake in *Saccharomyces cerevisiae*. J. Bacteriol..

[10] Ness F., Bourot S., Régnacq M., Spagnoli R., Bergès T., Karst F. (2001). *SUT1* is a putative Zn[II]_2_Cys_6_-transcription factor whose upregulation enhances both sterol uptake and synthesis in aerobically growing *Saccharomyces cerevisiae* cells. Eur. J. Biochem..

[11] Shianna K.V., Dotson W.D., Tove S., Parks L.W. (2001). Identification of a *UPC2* homolog in *Saccharomyces cerevisiae* and its involvement in aerobic sterol uptake. J. Bacteriol..

[12] Byrne K.P., Wolfe K.H. (2005). The Yeast Gene Order Browser: Combining curated homology and syntenic context reveals gene fate in polyploid species. Genome Res..

[13] Maguire S.L., Wang C., Holland L.M., Brunel F., Neuvéglise C., Nicaud J.M., Zavrel M., White T.C., Wolfe K.H., Butler G. (2014). Zinc finger transcription factors displaced SREBP proteins as the major Sterol regulators during Saccharomycotina evolution. PLoS Genet..

[14] Yang H., Tong J., Lee C.W., Ha S., Eom S.H., Im Y.J. (2015). Structural mechanism of ergosterol regulation by fungal sterol transcription factor Upc2. Nat. Commun..

[15] Rützler M., Reissaus A., Budzowska M., Bandlow W. (2004). *SUT2* is a novel multicopy suppressor of low activity of the cAMP/protein kinase A pathway in yeast. Eur. J. Biochem..

[16] Marie C., Leyde S., White T.C. (2008). Cytoplasmic localization of sterol transcription factors Upc2p and Ecm22p in *S. cerevisiae*. Fungal Genet. Biol..

[17] Lin M., Unden H., Jacquier N., Schneiter R., Just U., Höfken T. (2009). The Cdc42 effectors Ste20, Cla4, and Skm1 down-regulate the expression of genes involved in sterol uptake by a mitogen-activated protein kinase-independent pathway. Mol. Biol. Cell.

[18] Trendeleva T.A., Aliverdieva D.A., Zvyagilskaya R.A. (2014). Mechanisms of sensing and adaptive responses to low oxygen conditions in mammals and yeasts. Biochemistry.

[19] Henneberry A.L., Sturley S.L. (2005). Sterol homeostasis in the budding yeast, *Saccharomyces cerevisiae*. Semin. Cell Dev. Biol..

[20] Jacquier N., Schneiter R. (2012). Mechanisms of sterol uptake and transport in yeast. J. Steroid Biochem. Mol. Biol..

[21] Klug L., Daum G. (2014). Yeast lipid metabolism at a glance. FEMS Yeast Res..

[22] Bagnat M., Simons K. (2002). Cell surface polarization during yeast mating. Proc. Natl. Acad. Sci. USA.

[23] Heese-Peck A., Pichler H., Zanolari B., Watanabe R., Daum G., Riezman H. (2002). Multiple functions of sterols in yeast endocytosis. Mol. Biol. Cell.

[24] Tiedje C., Holland D.G., Just U., Höfken T. (2007). Proteins involved in sterol synthesis interact with Ste20 and regulate cell polarity. J. Cell Sci..

[25] Jin H., McCaffery J.M., Grote E. (2008). Ergosterol promotes pheromone signaling and plasma membrane fusion in mating yeast. J. Cell Biol..

[26] Aguilar P.S., Heiman M.G., Walther T.C., Engel A., Schwudke D., Gushwa N., Kurzchalia T., Walter P. (2010). Structure of sterol aliphatic chains affects yeast cell shape and cell fusion during mating. Proc. Natl. Acad. Sci. USA.

[27] Wilcox L.J., Balderes D.A., Wharton B., Tinkelenberg A.H., Rao G., Sturley S.L. (2002). Transcriptional profiling identifies two members of the ATP-binding cassette transporter superfamily required for sterol uptake in yeast. J. Biol. Chem..

[28] Li Y., Prinz W.A. (2004). ATP-binding cassette (ABC) transporters mediate nonvesicular, raft-modulated sterol movement from the plasma membrane to the endoplasmic reticulum. J. Biol. Chem..

[29] Gulati S., Balderes D., Kim C., Guo Z.A., Wilcox L., Area-Gomez E., Snider J., Wolinski H., Stagljar I., Granato J.T. (2015). ATP-binding cassette transporters and sterol *O*-acyltransferases interact at membrane microdomains to modulate sterol uptake and esterification. FASEB J..

[30] Abramova N.E., Cohen B.D., Sertil O., Kapoor R., Davies K.J., Lowry C.V. (2001). Regulatory mechanisms controlling expression of the *DAN*/*TIR* mannoprotein genes during anaerobic remodeling of the cell wall in *Saccharomyces cerevisiae*. Genetics.

[31] Alimardani P., Régnacq M., Moreau-Vauzelle C., Ferreira T., Rossignol T., Blondin B., Bergès T. (2004). *SUT1*-promoted sterol uptake involves the ABC transporter Aus1 and the mannoprotein Dan1 whose synergistic action is sufficient for this process. Biochem. J..

[32] Davies B.S., Rine J. (2006). A role for sterol levels in oxygen sensing in *Saccharomyces cerevisiae*. Genetics.

[33] Régnacq M., Alimardani P., El Moudni B., Bergès T. (2001). Sut1p interaction with Cyc8p(Ssn6p) relieves hypoxic genes from Cyc8p-Tup1p repression in *Saccharomyces cerevisiae*. Mol. Microbiol..

[34] Foster H.A., Cui M., Naveenathayalan A., Unden H., Schwanbeck R., Höfken T. (2013). The zinc cluster protein Sut1 contributes to filamentation in *Saccharomyces cerevisiae*. Eukaryot. Cell.

[35] Woods K., Höfken T. (2016). The zinc cluster proteins Upc2 and Ecm22 promote filamentation in *Saccharomyces cerevisiae* by sterol biosynthesis-dependent and -independent pathways. Mol. Microbiol..

[36] Kwast K.E., Lai L.C., Menda N., James D.T., Aref S., Burke P.V. (2002). Genomic analyses of anaerobically induced genes in *Saccharomyces cerevisiae*: Functional roles of Rox1 and other factors in mediating the anoxic response. J. Bacteriol..

[37] Luo Z., van Vuuren H.J. (2009). Functional analyses of *PAU* genes in *Saccharomyces cerevisiae*. Microbiology.

[38] Sertil O., Cohen B.D., Davies K.J., Lowry C.V. (1997). The *DAN1* gene of *S. cerevisiae* is regulated in parallel with the hypoxic genes, but by a different mechanism. Gene.

[39] Rachidi N., Martinez M.J., Barre P., Blondin B. (2000). *Saccharomyces cerevisiae PAU* genes are induced by anaerobiosis. Mol. Microbiol..

[40] Hickman M.J., Winston F. (2007). Heme levels switch the function of Hap1 of *Saccharomyces cerevisiae* between transcriptional activator and transcriptional repressor. Mol. Cell. Biol..

[41] Hickman M.J., Spatt D., Winston F. (2011). The Hog1 mitogen-activated protein kinase mediates a hypoxic response in *Saccharomyces cerevisiae*. Genetics.

[42] Inukai T., Nagi M., Morita A., Tanabe K., Aoyama T., Miyazaki Y., Bard M., Nakayama H. (2015). The mannoprotein *TIR3* (CAGL0C03872g) is required for sterol uptake in *Candida glabrata*. Biochim. Biophys. Acta.

[43] Abramova N., Sertil O., Mehta S., Lowry C.V. (2001). Reciprocal regulation of anaerobic and aerobic cell wall mannoprotein gene expression in *Saccharomyces cerevisiae*. J. Bacteriol..

[44] Espenshade P.J., Hughes A.L. (2007). Regulation of sterol synthesis in eukaryotes. Annu. Rev. Genet..

[45] Vik A., Rine J. (2001). Upc2p and Ecm22p, dual regulators of sterol biosynthesis in *Saccharomyces cerevisiae*. Mol. Cell. Biol..

[46] Germann M., Gallo C., Donahue T., Shirzadi R., Stukey J., Lang S., Ruckenstuhl C., Oliaro-Bosso S., McDonough V., Turnowsky F. (2005). Characterizing sterol defect suppressors uncovers a novel transcriptional signaling pathway regulating zymosterol biosynthesis. J. Biol. Chem..

[47] Valachovic M., Wilcox L.I., Sturley S.L., Bard M. (2004). A mutation in sphingolipid synthesis suppresses defects in yeast ergosterol metabolism. Lipids.

[48] Beh C.T., Cool L., Phillips J., Rine J. (2001). Overlapping functions of the yeast oxysterol-binding protein homologues. Genetics.

[49] Beh C.T., Rine J. (2004). A role for yeast oxysterol-binding protein homologs in endocytosis and in the maintenance of intracellular sterol-lipid distribution. J. Cell Sci..

[50] Davies B.S., Wang H.S., Rine J. (2005). Dual activators of the sterol biosynthetic pathway of *Saccharomyces cerevisiae*: Similar activation/regulatory domains but different response mechanisms. Mol. Cell. Biol..

[51] Hughes T.R., Marton M.J., Jones A.R., Roberts C.J., Stoughton R., Armour C.D., Bennett H.A., Coffey E., Dai H., He Y.D. (2000). Functional discovery via a compendium of expression profiles. Cell.

[52] Gallo-Ebert C., Donigan M., Liu H.Y., Pascual F., Manners M., Pandya D., Swanson R., Gallagher D., Chen W., Carman G.M. (2013). The yeast anaerobic response element AR1_b_ regulates aerobic antifungal drug-dependent sterol gene expression. J. Biol. Chem..

[53] Ter Linde J.J., Liang H., Davis R.W., Steensma H.Y., van Dijken J.P., Pronk J.T. (1999). Genome-wide transcriptional analysis of aerobic and anaerobic chemostat cultures of *Saccharomyces cerevisiae*. J. Bacteriol..

[54] MacIsaac K.D., Wang T., Gordon D.B., Gifford D.K., Stormo G.D., Fraenkel E. (2006). An improved map of conserved regulatory sites for *Saccharomyces cerevisiae*. BMC Bioinform..

[55] Lai L.C., Kosorukoff A.L., Burke P.V., Kwast K.E. (2006). Metabolic-state-dependent remodeling of the transcriptome in response to anoxia and subsequent reoxygenation in *Saccharomyces cerevisiae*. Eukaryot. Cell.

[56] Sertil O., Vemula A., Salmon S.L., Morse R.H., Lowry C.V. (2007). Direct role for the Rpd3 complex in transcriptional induction of the anaerobic *DAN/TIR* genes in yeast. Mol. Cell. Biol..

[57] Cohen B.D., Sertil O., Abramova N.E., Davies K.J., Lowry C.V. (2001). Induction and repression of *DAN1* and the family of anaerobic mannoprotein genes in *Saccharomyces cerevisiae* occurs through a complex array of regulatory sites. Nucleic Acids Res..

[58] Harbison C.T., Gordon D.B., Lee T.I., Rinaldi N.J., Macisaac K.D., Danford T.W., Hannett N.M., Tagne J.B., Reynolds D.B., Yoo J. (2004). Transcriptional regulatory code of a eukaryotic genome. Nature.

[59] Boyce K.J., Andrianopoulos A. (2011). Ste20-related kinases: Effectors of signaling and morphogenesis in fungi. Trends Microbiol..

[60] Lin M., Grillitsch K., Daum G., Just U., Höfken T. (2009). Modulation of sterol homeostasis by the Cdc42p effectors Cla4p and Ste20p in the yeast *Saccharomyces cerevisiae*. FEBS J..

[61] Montañés F.M., Pascual-Ahuir A., Proft M. (2011). Repression of ergosterol biosynthesis is essential for stress resistance and is mediated by the Hog1 MAP kinase and the Mot3 and Rox1 transcription factors. Mol. Microbiol..

[62] Saito H., Posas F. (2012). Response to hyperosmotic stress. Genetics.

[63] Cullen P.J., Sprague G.F. (2012). The regulation of filamentous growth in yeast. Genetics.

[64] Borneman A.R., Leigh-Bell J.A., Yu H., Bertone P., Gerstein M., Snyder M. (2016). Target hub proteins serve as master regulators of development in yeast. Genes Dev..

[65] Blanda C., Höfken T. (2013). Regulation of mating in the budding yeast *Saccharomyces cerevisiae* by the zinc cluster proteins Sut1 and Sut2. Biochem. Biophys. Res. Commun..

[66] Lorenz M.C., Heitman J. (1998). The MEP2 ammonium permease regulates pseudohyphal differentiation in *Saccharomyces cerevisiae*. EMBO J..

[67] Ryan O., Shapiro R.S., Kurat C.F., Mayhew D., Baryshnikova A., Chin B., Lin Z.Y., Cox M.J., Vizeacoumar F., Cheung D. (2012). Global gene deletion analysis exploring yeast filamentous growth. Science.

[68] Alvaro C.G., Thorner J. (2016). Heterotrimeric G Protein-coupled Receptor Signaling in Yeast Mating Pheromone Response. J. Biol. Chem..

[69] Atay O., Skotheim J.M. (2017). Spatial and temporal signal processing and decision making by MAPK pathways. J. Cell Biol..

[70] Burchett S.A., Scott A., Errede B., Dohlman H.G. (2001). Identification of novel pheromone-response regulators through systematic overexpression of 120 protein kinases in yeast. J. Biol. Chem..

[71] Butler G. (2013). Hypoxia and gene expression in eukaryotic microbes. Annu. Rev. Microbiol..

[72] Silver P.M., Oliver B.G., White T.C. (2004). Role of *Candida albicans* transcription factor Upc2p in drug resistance and sterol metabolism. Eukaryot. Cell.

[73] MacPherson S., Akache B., Weber S., de Deken X., Raymond M., Turcotte B. (2005). *Candida albicans* zinc cluster protein Upc2p confers resistance to antifungal drugs and is an activator of ergosterol biosynthetic genes. Antimicrob. Agents Chemother..

[74] Nagi M., Nakayama H., Tanabe K., Bard M., Aoyama T., Okano M., Higashi S., Ueno K., Chibana H., Niimi M. (2011). Transcription factors CgUPC2A and CgUPC2B regulate ergosterol biosynthetic genes in *Candida glabrata*. Genes Cells.

[75] Nocedal I., Johnson A.D. (2015). How Transcription Networks Evolve and Produce Biological Novelty. Cold Spring Harb. Symp. Quant. Biol..

[76] Tebung W.A., Choudhury B.I., Tebbji F., Morschhäuser J., Whiteway M. (2016). Rewiring of the Ppr1 Zinc Cluster Transcription Factor from Purine Catabolism to Pyrimidine Biogenesis in the Saccharomycetaceae. Curr. Biol..

[77] Pfaller M.A., Diekema D.J. (2007). Epidemiology of invasive candidiasis: A persistent public health problem. Clin. Microbiol. Rev..

[78] Höfken T., Kishore U., Nayak A. (2013). *Candida* and candidiasis. Microbial Pathogenesis.

[79] Znaidi S., Weber S., Al-Abdin O.Z., Bomme P., Saidane S., Drouin S., Lemieux S., de Deken X., Robert F., Raymond M. (2008). Genomewide location analysis of *Candida albicans* Upc2p, a regulator of sterol metabolism and azole drug resistance. Eukaryot. Cell.

[80] Hoot S.J., Oliver B.G., White T.C. (2008). *Candida albicans UPC2* is transcriptionally induced in response to antifungal drugs and anaerobicity through Upc2p-dependent and -independent mechanisms. Microbiology.

[81] Hoot S.J., Brown R.P., Oliver B.G., White T.C. (2010). The UPC2 promoter in *Candida albicans* contains two *cis*-acting elements that bind directly to Upc2p, resulting in transcriptional autoregulation. Eukaryot. Cell.

[82] Dunkel N., Liu T.T., Barker K.S., Homayouni R., Morschhäuser J., Rogers P.D. (2008). A gain-of-function mutation in the transcription factor Upc2p causes upregulation of ergosterol biosynthesis genes and increased fluconazole resistance in a clinical *Candida albicans* isolate. Eukaryot. Cell.

[83] Heilmann C.J., Schneider S., Barker K.S., Rogers P.D., Morschhäuser J. (2010). An A643T mutation in the transcription factor Upc2p causes constitutive *ERG11* upregulation and increased fluconazole resistance in *Candida albicans*. Antimicrob. Agents Chemother..

[84] Hoot S.J., Smith A.R., Brown R.P., White T.C. (2011). An A643V amino acid substitution in Upc2p contributes to azole resistance in well-characterized clinical isolates of *Candida albicans*. Antimicrob. Agents Chemother..

[85] Flowers S.A., Barker K.S., Berkow E.L., Toner G., Chadwick S.G., Gygax S.E., Morschhäuser J., Rogers P.D. (2012). Gain-of-function mutations in *UPC2* are a frequent cause of *ERG11* upregulation in azole-resistant clinical isolates of *Candida albicans*. Eukaryot. Cell.

[86] Synnott J.M., Guida A., Mulhern-Haughey S., Higgins D.G., Butler G. (2010). Regulation of the hypoxic response in *Candida albicans*. Eukaryot. Cell.

[87] Sasse C., Dunkel N., Schäfer T., Schneider S., Dierolf F., Ohlsen K., Morschhäuser J. (2012). The stepwise acquisition of fluconazole resistance mutations causes a gradual loss of fitness in *Candida albicans*. Mol. Microbiol..

[88] Lohberger A., Coste A.T., Sanglard D. (2014). Distinct roles of *Candida albicans* drug resistance transcription factors *TAC1*, *MRR1*, and *UPC2* in virulence. Eukaryot. Cell.

[89] Vandeputte P., Ischer F., Sanglard D., Coste A.T. (2011). *In vivo* systematic analysis of *Candida albicans* Zn2-Cys6 transcription factors mutants for mice organ colonization. PLoS ONE.

[90] Zavrel M., Hoot S.J., White T.C. (2013). Comparison of sterol import under aerobic and anaerobic conditions in three fungal species, *Candida albicans*, *Candida glabrata*, and *Saccharomyces cerevisiae*. Eukaryot. Cell.

[91] Sudbery P.E. (2011). Growth of *Candida albicans* hyphae. Nat. Rev. Microbiol..

[92] Gow N.A., van de Veerdonk F.L., Brown A.J., Netea M.G. (2011). *Candida albicans* morphogenesis and host defence: Discriminating invasion from colonization. Nat. Rev. Microbiol..

[93] Gallo-Ebert C., Donigan M., Stroke I.L., Swanson R.N., Manners M.T., Francisco J., Toner G., Gallagher D., Huang C.Y., Gygax S.E. (2014). Novel antifungal drug discovery based on targeting pathways regulating the fungus-conserved Upc2 transcription factor. Antimicrob. Agents Chemother..

[94] Xu W., Solis N.V., Ehrlich R.L., Woolford C.A., Filler S.G., Mitchell A.P. (2015). Activation and alliance of regulatory pathways in *C. albicans* during mammalian infection. PLoS Biol..

[95] Nakayama H., Izuta M., Nakayama N., Arisawa M., Aoki Y. (2000). Depletion of the squalene synthase (*ERG9*) gene does not impair growth of *Candida glabrata* in mice. Antimicrob. Agents Chemother..

[96] Bard M., Sturm A.M., Pierson C.A., Brown S., Rogers K.M., Nabinger S., Eckstein J., Barbuch R., Lees N.D., Howell S.A. (2005). Sterol uptake in *Candida glabrata*: Rescue of sterol auxotrophic strains. Diagn. Microbiol. Infect. Dis..

[97] Nagi M., Tanabe K., Nakayama H., Yamagoe S., Umeyama T., Oura T., Ohno H., Kajiwara S., Miyazaki Y. (2013). Serum cholesterol promotes the growth of *Candida glabrata* in the presence of fluconazole. J. Infect. Chemother..

[98] Whaley S.G., Caudle K.E., Vermitsky J.P., Chadwick S.G., Toner G., Barker K.S., Gygax S.E., Rogers P.D. (2014). *UPC2A* is required for high-level azole antifungal resistance in *Candida glabrata*. Antimicrob. Agents Chemother..

[99] Nakayama H., Tanabe K., Bard M., Hodgson W., Wu S., Takemori D., Aoyama T., Kumaraswami N.S., Metzler L., Takano Y. (2007). The *Candida glabrata* putative sterol transporter gene *CgAUS1* protects cells against azoles in the presence of serum. J. Antimicrob. Chemother..

[100] Silva A.P., Miranda I.M., Guida A., Synnott J., Rocha R., Silva R., Amorim A., Pina-Vaz C., Butler G., Rodrigues A.G. (2011). Transcriptional profiling of azole-resistant *Candida parapsilosis* strains. Antimicrob. Agents Chemother..

[101] Guida A., Lindstädt C., Maguire S.L., Ding C., Higgins D.G., Corton N.J., Berriman M., Butler G. (2011). Using RNA-seq to determine the transcriptional landscape and the hypoxic response of the pathogenic yeast *Candida parapsilosis*. BMC Genom..

[102] Berkow E.L., Manigaba K., Parker J.E., Barker K.S., Kelly S.L., Rogers P.D. (2015). Multidrug Transporters and Alterations in Sterol Biosynthesis Contribute to Azole Antifungal Resistance in *Candida parapsilosis*. Antimicrob. Agents Chemother..

[103] Choi M.J., Won E.J., Shin J.H., Kim S.H., Lee W.G., Kim M.N., Lee K., Shin M.G., Suh S.P., Ryang D.W. (2016). Resistance Mechanisms and Clinical Features of Fluconazole-Nonsusceptible *Candida tropicalis* Isolates Compared with Fluconazole-Less-Susceptible Isolates. Antimicrob. Agents Chemother..

[104] Jiang C., Ni Q., Dong D., Zhang L., Li Z., Tian Y., Peng Y. (2016). The Role of *UPC2* Gene in Azole-Resistant *Candida tropicalis*. Mycopathologia.

